# Supplementary material for: A Comprehensive Assessment of Ultraviolet-Radiation-Induced Mutations in Flammulina filiformis Using Whole-Genome Resequencing
Source: J Fungi (Basel). 2024 Mar 20;10(3):228. doi: 10.3390/jof10030228 (PMC10971301; doi:10.3390/jof10030228)
Supplement: Supplementary file 1 [file jof-10-00228-s001.zip › Supplementary Material S8/KEGG annotation/out/64381550635650.os/KO/out_map/map05166.html]

KEGG PATHWAY: HTLV-I infection - Reference pathway


|  |  |
| --- | --- |
| **HTLV-I infection - Reference pathway** |  |

[
Pathway menu
| Organism menu
| Pathway entry
| Show description
| User data mapping
]

Human T-lymphotropic virus type 1 (HTLV-1) is a pathogenic retrovirus that is associated with adult T-cell leukemia/lymphoma (ATL). It is also strongly implicated in non-neoplastic chronic inflammatory diseases such as HTLV-1-associated myelopathy/tropical spastic paraparesis (HAM/TSP). Expression of Tax, a viral regulatory protein is critical to the pathogenesis. Tax is a transcriptional co-factor that interfere several signaling pathways related to anti-apoptosis or cell proliferation. The modulation of the signaling by Tax involve its binding to transcription factors like CREB/ATF, NF-kappa B, SRF, and NFAT.

|  |  |
| --- | --- |
| Reference pathway | 100% |
